# Mechanical Circulatory Support with Impella: Principles, Evidence, and Daily Practice

**DOI:** 10.3390/jcm13164586

**Published:** 2024-08-06

**Authors:** Giulia Masiero, Federico Arturi, Andrea Panza, Giuseppe Tarantini

**Affiliations:** Department of Cardiac, Thoracic, Vascular Sciences and Public Health, University of Padua Medical School, 35128 Padua, Italy; giulia.masiero.86@gmail.com (G.M.); federicoarturi@gmail.com (F.A.); andreapanza93@gmail.com (A.P.)

**Keywords:** cardiogenic shock, high-risk percutaneous coronary intervention, mechanical circulatory support, percutaneous left ventricular assist devices

## Abstract

The Impella (Abiomed, Danvers, MA, USA) microaxial pump is a percutaneous mechanical circulatory support (MCS) that has been shown to increase coronary perfusion, reduce myocardial oxygen demand, and improve peripheral organ perfusion. Therefore, indications for the Impella device include emergency use for cardiogenic shock (CS) and pre-emptive implantation during high-risk percutaneous coronary intervention (HR-PCI). However, despite their exponential use in cardiovascular practice over the past decade, there is limited randomized evidence to support the benefits of this therapy and growing concern regarding complication rates. In this review, we summarize the principles, evidence, and practical considerations of the most widely used Impella CP percutaneous left ventricular support in both CS and HR-PCI settings, moving from the historical background to current issues and future expectations for this device.

## 1. Introduction

In recent years, there has been a notable rise in the use of temporary mechanical circulatory support (MCS), such as percutaneous left ventricular assist devices (pLVADs) and extracorporeal veno-arterial membrane oxygenation (VA-ECMO). This trend aims to enhance outcomes in cases of cardiogenic shock (CS) and complex percutaneous coronary intervention (PCI). Among the available percutaneous supports, the intra-aortic balloon pump (IABP) has not shown a clear clinical advantage compared to conventional treatment [1]. For this reason, IABP usage has seen a significant reduction through the years, even though its employment is still not negligible [2]. Additionally, the commercially available Impella family of MCS (Abiomed, Danvers, Massachusetts, USA) has emerged as another significant player in this field.

The Impella system consists of a catheter-based microaxial continuous flow device placed across the aortic valve actively pumping blood from the left ventricle (LV) into the ascending aorta throughout the cardiac cycle, regardless of the heart’s rhythm and cardiac function. It comprises various models with different unloading power and supported flow, including the Impella 2.5 (up to 2.5 L/min; 13F), Impella CP (with Smart Assist, 3.5 to 4.3 L/min; 14F), and Impella 5.0 and 5.5 (with Smart Assist, up to 6.2 L/min; 23F), with an additional RP model available for right ventricular support (4.0 L/min; 23F). While the 2.5 and CP devices can be percutaneously inserted, the 5.0 and 5.5 require a surgical approach for transfemoral, transaxillary, or trans-subclavian implantation. Currently, Impella CP, 5.5, and RP are the only commercially available devices. The Smart Assist system, integrated into recent models, uses real-time intelligence to optimize support positioning, management, and weaning. By employing LV-to-aorta support, the Impella support reduces ventricular workload, enhances cardiac output, and facilitates myocardial recovery in cardiogenic shock (CS) [3,4]. Moreover, the pump improves distal coronary flow and coronary perfusion pressure, potentially reducing myocardial injury during high-risk PCI (HR-PCI) [5,6].

Moving on from the historical background of the LV microaxial pump, this review will focus on the most widely used Impella CP percutaneous LV support in both CS and non-emergent complex high-risk indicated procedure (CHIP) settings. It will progress from analyzing available evidence to providing practical recommendations for optimal practice.

## 2. Use in High-Risk PCI

HR-PCI is characterized by the presence of reduced LV ejection fraction (LVEF), critically elevated LVEDP, and high-risk anatomical features of coronary artery disease (CAD), such as unprotected left main disease, multivessel disease, chronic total occlusion, severely calcified lesions requiring debulking, complex bifurcation lesions, last patent conduit, and degenerated saphenous vein grafts [6,7]. In recent decades, PCI has emerged as the preferred revascularization approach for a growing population of patients with non-emergent complex CAD. This is particularly true for individuals considered unsuitable candidates for cardiac surgery or who, after informed consent, opt against coronary artery bypass graft surgery [8]. Given that these procedures typically involve advanced technologies and extended durations, the use of hemodynamic support with Impella has become a preventive measure. It aims to mitigate the expected risk of hemodynamic instability resulting from diminished coronary flow and transient ischemia following balloon and stent inflation, debulking system usage, contrast medium injection, and potential occlusion or dissection induced by guiding catheters [6].

Conclusive evidence of a clinical benefit of CHIP preventive support has not yet been demonstrated. Nevertheless, its real-world utilization is expanding, supported by non-randomized data indicating a potential decrease in adverse events and functional improvement. Evidence supporting the use of Impella in HR-PCI scenarios is derived primarily from a single randomized controlled trial (RCT), selective post hoc analyses, and large registries or matched analysis (Table 1).

The Prospective, Multicenter Randomized Controlled (PROTECT) II trial compared the outcomes of assisted HR-PCI using the Impella 2.5 or IABP [9]. Despite its premature termination by the Data Safety Monitoring Board after an interim analysis suggested futility, the trial encompassed a total of 452 patients undergoing non-emergent HR-PCI, characterized by complex CAD and impaired LVEF. While the rates of major adverse events (MAEs) at 30 days were comparable between the two groups (34% vs. 42%, *p* = 0.092), they were significantly lower at 90 days in the Impella group according to per-protocol analysis (40% vs. 51%, *p* = 0.023) and when restricting the intention-to-treat analysis to patients with 3-vessel CAD and LVEF ≤30% (39.5% vs. 51.0%, *p* = 0.039) [10]. Another post hoc analysis using a higher cardiac biomarker threshold confirmed a significant 90-day reduction in major adverse cardiac and cerebrovascular events (MACCEs) in the Impella group patients [11].

Patients from the largest retrospective cVAD registry meeting the PROTECT II study criteria were compared to the original Impella arm of the RCT [8,12]. Despite being older with more comorbidities and a higher likelihood of prior PCI and extensive coronary disease, they achieved more complete revascularization and experienced lower rates of in-hospital mortality, myocardial infarction (MI), and repeat revascularization. Subsequent registries further supported these findings, demonstrating that more complete revascularization was associated with significant improvement in LVEF at mid-term follow-up after Impella-assisted PCI [13,14]. Recently, a sensitivity analysis of individual patient data from the PROTECT II and RESTORE-EF trials highlighted that higher baseline LVEF, a lower residual Synergy Between Percutaneous Coronary Intervention with Taxus and Cardiac Surgery (SYNTAX) score, and Impella treatment were independent predictors of LVEF improvement at 90 days post-PCI in patients with stable and complex CAD and severe LVEF reduction [15]. Furthermore, a propensity-weighted analysis from a large Italian registry suggested a survival benefit and reduced rates of major bleeding when Impella implantation was performed pre-PCI instead of during or after the procedure in a CHIP setting [16,17]. Additionally, Impella support carried a minor risk of developing post-procedural acute kidney injury (AKI) compared to the predicted rate of renal dysfunction calculated according to the Mehran risk score, as per a sub-analysis from the cVAD registry [18].

Despite the increasing use of MCS in CHIP settings, there is still a lack of comparative real-world data determining the superiority of one device over another or over optimal medical therapy alone. A matched cohort study of 500 patients undergoing high-risk PCI with or without pLVAD support found higher in-hospital MACCE rates in the Impella group, largely attributed to more aggressive procedures with a higher incidence of periprocedural MI, major bleeding, and the need for blood transfusions [19]. However, no differences were observed in composite outcomes at 1-year follow-up. Conversely, a propensity-weighted analysis including 2156 patients undergoing non-emergent HR-PCI with percutaneous support demonstrated improved survival in the Impella cohort, with no differences in terms of AKI, bleeding requiring transfusion, or stroke compared to the IABP group [20]. Compared to VA-ECMO, two small observational studies revealed no significant difference in 30-day mortality and MACE rate among patients supported by Impella. However, Impella-supported patients exhibited a lower risk of post-procedural AKI [20,21,22].

**Table 1:** Randomized controlled trials, observational registries, and matched/adjusted analyses regarding Impella use in high-risk PCI.

Name	Year	Setting	N	Intervention vs. Control	Endpoints	Results
**Randomized controlled trial**						
**O’Neill et al. [8]**	2012	Non-emergent HR-PCI	452	Impella 2.5 vs. IABP	30- and 90-day MAE	Non-inferiority
**Observational registry**						
**Cohen et al. [11]**	2015	Non-emergent HR-PCI	555	Impella 2.5	In-hospital MAE	Comparable with P-II pts
**Burzotta et al. [12]**	2019	Non-emergent HR-PCI	79	Impella 2.5/CP	LVEF at 180 days	+26%
**Baumann et al. [22]**	2019	Non-emergent HR-PCI	157	Impella 2.5/CP	6-month MACE	22.8%
**Chieffo et al. [15]**	2020	Non-emergent HR-PCI	177	Impella 2.5/CP	1-year all-cause death	15.6%
**Wollmuth et al. [13]**	2022	Non-emergent HR-PCI	251	Impella 2.5/CP	LVEF at 90 days	+29%
**Matched/adjusted analysis**						
**Kovacic et al. [9]**	2015	Non-emergent HR-PCI	325	Impella 2.5 vs. IABP	90-day MAE	Impella was better
**Azzalini et al. [18]**	2020	Non-emergent HR-PCI	474	Impella 2.5/CP vs. no MCS	1 year MACE	No differences
**Lansky et al. [19]**	2022	Non-emergent HR-PCI	2156	Impella 2.5/CP vs. IABP	In-hospital mortality	Impella was better
**Van den Buijs et al. [20]**	2022	Non-emergent HR-PCI	41	Impella CP vs. VA-ECMO	30-day mortality	No differences
**Panoulas et al. [14]**	2024	Non-emergent HR-PCI	344	Impella 2.5/CP vs. IABP	LVEF at 90 days	Impella was better

ECMO, extracorporeal membrane oxygenation; IABP, intra-aortic balloon pump; HR-PCI, high-risk percutaneous coronary intervention; LVEF, left ventricular ejection fraction; MAE, major adverse event, MACE, major adverse cardiovascular event; MCS, mechanical circulatory support.

## 3. Use in CS

CS results from inadequate cardiac output due to severe cardiac dysfunction, leading to persistent tissue hypoperfusion and multi-organ failure [23]. Despite advancements in standard medical treatment, CS continues to carry high morbidity and mortality rates [24]. In this context, the Impella device has gained prominence for its ability to directly unload the LV, reducing cardiac workload and pulmonary capillary wedge pressure while improving forward flow, systemic pressure, and perfusion.

Although MCS utilization in CS is increasing, the quality of evidence supporting its use remains a topic of debate. Presently, four RCTs have explored Impella’s efficacy in CS, with conflicting findings from various observational studies (Table 2).

The ISAR-SHOCK trial, comparing Impella 2.5 with IABP in CS due to acute MI, demonstrated a significant increase in cardiac index post-Impella implantation compared to IABP. However, no difference in 30-day mortality was noted [25]. Similarly, the IMPRESS trial found no significant differences in early mortality rates between microaxial pLVAD and IABP-treated MI-CS patients [26]. Moreover, the IMPELLA-STIC trial, adding Impella 5.0 to initial inotrope and IABP treatment in MI-CS patients, showed no significant changes in baseline cardiac output index until 12 h post-treatment, with major bleeding events more common in the Impella 5.0 group [27]. Conversely, the DanGer Shock trial, randomizing ST-elevation myocardial infarction (STEMI) CS patients to standard care alone or with Impella CP, demonstrated a survival benefit in the Impella group at 180-day follow-up, albeit with a increased occurrence of safety composite endpoint, primarily due to augmented bleeding, renal replacement, and sepsis rates [28]. It randomized 360 patients with STEMI complicated by Society for Cardiovascular Angiography and Interventions (SCAI) shock stages C, D, and E, excluding patients with out-of-hospital cardiac arrest who remained comatose and those with right ventricular failure. At the 180-day follow-up, the Impella CP group had a lower mortality rate compared to the standard care group (HR 0.74, 95% CI 0.55–0.99; *p* = 0.04). However, the Impella group had a higher safety composite endpoint compared to the standard care group [28]. A pre-specified sub-analysis suggested a greater survival benefit in patients with a lower mean arterial pressure (≤63 mm Hg) and those with multivessel disease. Overall, these results are significant as they represent the first treatment strategy to demonstrate a survival benefit in patients with acute MI complicated by CS since the SHOCK trial in 1999 [29] and mark a significant departure from the negative outcomes observed in previous trials involving other MCS [1,30]. The trial’s success underscores the importance of meticulous patient selection, early device placement before reperfusion, safe vascular access and closure techniques, and the application of standardized weaning and removal protocols in optimizing outcomes for patients with acute myocardial infarction complicated by cardiogenic shock. 

Large observational analyses indicated higher survival rates with the placement of pLVADs before irreversible metabolic impairment and prior to PCI to ensure extensive coronary revascularization [31]. Specifically, the preventive use of Impella CP has demonstrated reductions in vascular complications and early hazards associated with complete revascularization [17,32]. Furthermore, Schrage et al. underscore the importance of early LV unloading, showing a lower 30-day mortality in CS patients treated with VA-ECMO in combination with an Impella device compared to with VA-ECMO alone, despite increased bleeding and ischemic complications [33]. Several registries have shifted focus to the clinical hazards associated with the use of MCS devices with large sheaths, particularly in the emergent setting of acute MI complicated by CS, reporting an elevated risk of severe or life-threatening bleeding and peripheral vascular complications [34,35,36,37,38,39,40].

**Table 2:** Major randomized controlled trials and retrospective matched/adjusted analysis regarding Impella use in cardiogenic shock.

Name	Year	Setting	N	Intervention vs. Control	Endpoints	Results
**Randomized controlled trial**						
**ISAR-Shock [25]**	2008	CS	26	Impella LP2.5 vs. IABP	Change in CI after 30 min	Impella was better
**IMPRESS [26]**	2017	CS-AMI (STEMI)	48	Impella vs. IABP	30-day all-cause mortality	No differences
**IMPELLA-STIC [27]**	2020	CS-AMI (STEMI)	12	Impella LP 5.0 vs. IABP	Change in CPI after 12 h	No differences
**DANGER SHOCK [28]**	2024	CS-AMI (STEMI)	360	Impella CP vs. standard of care	180-day all-cause mortality	Impella was better
**Retrospective matched/adjusted analysis**						
**IABP-SHOCK II [35]**	2019	CS-AMI (N/STEMI)	474	Impella vs. entire cohort of IABP-SHOCK II	30-day all-cause mortality	No differences
**IABP-SHOCK II [35]**	2019	CS-AMI (N/STEMI)	230	Impella vs. IABP cohort of IABP-SHOCK II	30-day all-cause mortality	No differences
**Karami et al. [40]**	2020	CS	128	Impella CP/5.0 vs. ECMO	30-day all-cause mortality	No differences
**Schrage et al. [33]**	2020	ECLS-treated CS	510	VA ECMO and Impella vs. VA ECMO	30-day all-cause mortality	Impella was better
**Dhruva et al. [36]**	2020	CS-AMI (N/STEMI)	3360	Impella vs. IABP	In-hospital all-cause death	pLVAD was worse
**Scherer et al. [39]**	2020	CS	140	Impella CP vs. no ELCS	1-year and 5-year all-cause mortality	No differences
**Wernly et al. [37]**	2021	CS	149	Impella 2.5 vs. ECLS	30-day all-cause mortality	No differences
**Sieweke et al. [38]**	2021	rCS after OHCA	30	Impella vs. standard of care	30-day all-cause mortality	Impella was better

CS, cardiogenic shock; AMI, acute myocardial infarction; ECLS, extracorporeal life support; rCS, refractory cardiogenic shock; OHCA, out-of-hospital cardiac arrest; CPI, cardiac power index; CI, cardiac index; IABP, intra-aortic balloon pump; ECMO, extracorporeal membrane oxygenation.

## 4. Adverse Events

As previously demonstrated, bleeding and vascular complications represent significant challenges associated with the use of microaxial pLVADs, strongly linked to increased mortality, prolonged hospital stays, and higher costs [41]. Factors such as large-bore access, the necessity for anticoagulation, shock-induced coagulation changes, and device-related mechanical shear stress contribute to the development of adverse hemorrhagic and ischemic events. In the Italian IMP-IT Registry, device-related complications (DRCs) occurred in a quarter of the population, with a higher incidence observed in the CS group compared to the HR-PCI population [42]. CS presentation, low EF, and peripheral artery disease emerged as strong and independent predictors of DRCs, while the use of preclosure devices appeared to be protective. Common complications included hemolysis, access-site bleeding, and limb ischemia. Furthermore, CS and pulmonary hypertension are risk factors for hemolysis, which, in turn increases the risk of AKI and the need for renal replacement therapy. Conversely, female sex, advanced age, and Impella 5.0 usage are associated with vascular bleeding and complications. The use of microaxial pLVADs carries a higher risk of complications, including hemolysis, major bleeding, ischemic vascular complications, and stroke, compared to IABP use, although not when compared to VA-ECMO, in both CS and HR-PCI settings [26,28,34,35,36]. Notably, patients enrolled in the cVAD registry showed a lower rate of mid-term MACCE and bleeding requiring transfusions compared to the initial PROTECT II experience, despite having a higher frailty profile and undergoing more complex procedures. This underscores the significant role of the learning curve with the device and the widespread adoption of standard procedures in vascular access management [43].

## 5. Best Practice

**Figure 1:**
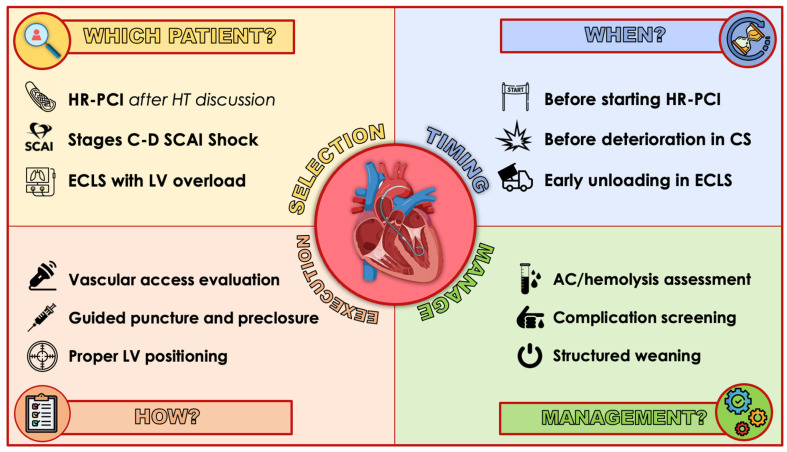
Suggested best practice in percutaneous Impella CP use. HR-PCI, high-risk PCI; HT, heart team; CS, cardiogenic shock; SCAI, Society for Cardiovascular Angiography and Interventions; ECMO, extracorporeal veno-arterial membrane oxygenation; LV, left ventricle; AC, anticoagulation.

All the principles outlined in this section are applicable to the use of the Impella CP device in both CS and HR-PCI settings, unless specifically indicated otherwise (Figure 1). 

### 5.1. Patient and Device Selection

The decision to use Impella typically involves a multidisciplinary discussion. In HR-PCI, following the determination of high-risk status, patients should undergo evaluation by the local heart team, comprised of interventionists, cardiac surgeons, and anesthesiologists. Based on factors such as anatomy, comorbidities, and the risk of hemodynamic compromise, the team will determine the optimal revascularization strategy and whether MCS is necessary [44,45]. Similarly, in the context of CS, a standardized team-based approach has been shown to yield improved outcomes [46]. Recognition of the SCAI shock stages (C to E) is crucial, with MCS initiation beyond vasoactive agents often required after careful evaluation of patient characteristics, including the illness acuity, CS phenotype, and vascular access anatomy. Impella CP is typically employed in SCAI stages C or D when LV support is needed, while its use in high-risk acute MI with SCAI stage B as a preventive unloading strategy is currently under investigation. In cases of SCAI shock at stage E, cardiac arrest, biventricular, or pulmonary failure, VA-ECMO may be a more suitable choice [6].

### 5.2. Timing

Early implantation appears to be a critical factor that can positively influence outcomes, whether it involves pre-PCI insertion, early unloading in patients undergoing VA-ECMO therapy, or prompt usage in CS before irreversible metabolic impairment occurs [33,47,48].

### 5.3. Access

Proper access management is essential in reducing vascular complications associated with microaxial pLVADs. Tools such as ultrasound Doppler, computed tomography angiography, or arteriography during emergent procedures aid in appropriate access site selection by assessing vascular tortuosity and calcifications and ensuring diameters of at least 5 mm for Impella CP [49,50,51]. Femoral access is preferred in most cases and can be obtained either percutaneously (Impella CP) or through surgical cut-down (Impella 5.0). Contemporary puncture approaches, involving echo and fluoroscopic evaluation or micropuncture assessment, have been shown to reduce adverse events [52]. Techniques like long sheaths, balloon angioplasty, vascular stenting, and intravascular lithotripsy are useful in cases of tortuous or calcified ileo-femoral axes. Transaxillary and subclavian arteries are alternative vascular options, particularly when a longer duration of support or larger device sizes are anticipated [50,53]. While preclosure management is recommended, there is no definitive superiority between suture-based and plug-based devices [54,55]. The single-access approach has gained popularity to minimize unnecessary vascular access, with the difference in sizes between the Impella sheath and driver shaft allowing for the placement of standard 6Fr or hydrophilic-coated 7Fr sheaths. For larger lumen catheters, using an 8 Fr guiding catheter delivered sheathless using Rotaglide is a feasible solution [56].

### 5.4. Device Positioning

After gaining access to the vascular lumen, the Impella sheath is advanced through a stiff guidewire, by crossing the aortic valve (AV) with a pigtail catheter and then exchanging it with a 0.018″ wire. Following anticoagulation administration with a target activated clotting time (ACT) ≥ 250 s, the 0.018″ guidewire is inserted into a removable monorail small pipe to facilitate retrograde Impella advancement up to the LV. The malpositioning of the Impella through the AV may lead to inadequate support, hemolysis, and iatrogenic mechanical complications [57,58]. Assessing Impella location in the catheterization laboratory is primarily achieved via fluoroscopy. A guidewire or pigtail catheter in the non-coronary sinus can aid in highlighting the AV position if any doubts arise [59]. Bedside echocardiography is essential to measure the distance between the AV and Impella’s inlet cage, ideally around 3.5 cm, and to guide adjustments [59]. The microaxial pLVAD should ideally sit free from the mitral valvular and subvalvular apparatus, with the pump in the mid-LV cavity, the tip pointing towards the LV apex, and the outflow above the aortic valve. The device can confirm correct positioning by detecting pressure variations across the input and output ports. However, in cases of severe LV impairment and ventriculo-arterial uncoupling, pulsatility may be lacking even with correct device positioning [60]. With the addition of an optical sensor and SmartAssist technology, Impella can now analyze both aortic and LV pressure waveforms. Inadequate preload, due to factors like hypovolemia, right ventricular failure, or vasoplegia, may result in a negative diastolic pressure with normal systolic pressure in the LV pressure trace. Both low diastolic and systolic pressures indicate Impella malposition and suction phenomena, a major cause of hemolysis [57,60]. Hemolysis correlates with flow rate, increasing as Impella’s rotations per minute increase [61], while lower flow rates are associated with increased ischemic risk.

### 5.5. Anticoagulation

Anticoagulation, administered systemically and through a purge solution, is essential to counteract device-related mechanical shear stress and pump contact with blood, which are potent clotting activators. Systemic anticoagulation is usually maintained through the continuous infusion of unfractioned heparin (UFH), while bivalirudin and argatroban are preferred in cases of heparin-induced thrombocytopenia (HIT). The purge solution is a mixture of dextrose and UFH or bivalirudin/argatroban in the case of HIT. Both dextrose 5% (D5%) and dextrose 20% (D20%) can be used; the less viscous the solution, the higher the flow (approximately 30–40% higher with D5% than with D20%). The flow rate is automatically determined by the device and, with it, the amount of UFH delivered via the purge system. Changing the viscosity of the solution hence is a way to (partially) influence the administered heparin dosage. In cases of severe bleeding and absolute contraindication to anticoagulation, purge solutions based on bicarbonate have been reported [60].

### 5.6. Bleeding Management

Prevention is paramount, but in cases of bleeding, the initial step is to control the source. For vascular access bleeding, manual compression at the puncture site, along with local application of tranexamic acid/adrenaline-soaked gauzes (1:100 concentration), can often suffice to manage oozing. Gastrointestinal, respiratory, and urinary bleeding typically requires endoscopy for source control, with the cauterization of bleeding lesions [62]. Lowering or discontinuing anticoagulation should be considered only when acceptable source control cannot be achieved or major surgical interventions are planned for severe intracranial or retroperitoneal bleeding. Initially, systemic anticoagulation levels are reduced; if this is insufficient, then shifting to a bicarbonate-based purge solution may be considered. Whenever anticoagulation levels are reduced, the pump speed should be maximized to mitigate ischemic events. In cases of major bleedings, weaning from microaxial pLVAD should be evaluated. The use of reversal agents (such as protamine, fresh frozen plasma, prothrombin complex concentrate, or intravenous tranexamic acid) should be reserved for life-threatening bleeding situations after device removal.

### 5.7. Daily Monitoring

In cases of prolonged support, comprehensive daily monitoring is recommended. This includes physical examinations, neurological assessments, and examinations of limbs and vascular access. Regular checks of blood counts and plasma free hemoglobin (pfHb), lactate dehydrogenase, bilirubin, and haptoglobin levels are essential. According to recent guidelines, pfHb levels exceeding 20 mg/dL indicate hemolysis [63]. Coagulation status should be assessed frequently, with activated partial thromboplastin time and heparin anti-factor Xa levels monitored every 3–6 h. The daily monitoring of fibrinogen, international normalized ratio (INR), antithrombin III, and D-Dimer is recommended, especially if intravascular disseminated coagulation, heparin resistance, or thrombosis is suspected. Bedside echocardiography should be conducted daily to confirm device positioning, detect malfunction causes (such as malposition or low filling volume due to right ventricular failure/hypovolemia), and assess LV function and dimensions. This evaluation, along with the exclusion of mechanical or valvular complications, serves as the basis for the weaning process. Although no RCTs have demonstrated the benefits of invasive Swan–Ganz monitoring in the intensive care unit, a large meta-analysis of observational studies indicated a short-term survival benefit in patients with cardiogenic shock who underwent pulmonary artery catheter monitoring [64]. Therefore, its use can offer valuable insights into filling status, LV pressures, and right ventricular function, aiding in troubleshooting and the weaning process.

### 5.8. Weaning

In elective HR-PCI without hemodynamic complications, rapid weaning protocols are often recommended. Initially, support is swiftly reduced to P2 levels, followed by further lowering to P1 after confirming hemodynamic stability. The device is then removed after being completely turned off in the descending aorta [65]. For cases requiring prolonged support, weaning recommendations rely on expert consensus. A small retrospective cohort study indicated that higher creatinine and lactate levels at Impella insertion, as well as the magnitude of inotropic support at time of weaning, were associated with unsuccessful weaning attempts. Additionally, a low ejection fraction (EF) and lactate elevation post-weaning were independent predictors of post-removal mortality [66]. Weaning attempts are typically made in stable patients with lactate levels below 2.0 mmol/L, the absence of mechanical or valvular complications, a heart rate between 60 and 100 bpm, and no signs of pulmonary congestion (preferably with a pulmonary capillary wedge pressure <15 mmHg). The mean arterial pressure should ideally exceed 65 mmHg with a pulsatile waveform and minimal inotropic support. During the weaning process, the flow rate is ideally halved for 4–8 h, with ongoing clinical assessment. If no deterioration occurs, further reduction in microaxial pump power is initiated, followed by removal after another 4–8 h of monitoring. Any clinical deterioration prompts immediate support escalation, with meticulous attention to anticoagulation [6].

## 6. Gaps in Knowledge

Several RCTs are currently underway to address the ongoing debate regarding the effectiveness of Impella devices, not only in terms of improving hemodynamics but also in improving clinical outcomes compared to standard medical therapy or other MCS devices. In the context of high-risk STEMI, the STEMI DTU trial aims to evaluate the impact of mechanical LV unloading with Impella devices compared to standard medical therapy before primary PCI [67]. The goal is to determine whether reducing wall stress and oxygen consumption with Impella support can decrease the size of the infarct area and mitigate ischemia–reperfusion injury. Additionally, the ULYSS and RECOVER IV studies seek to further elucidate the potential benefits of adding Impella support to standard medical therapy in AMI-CS patients undergoing PCI [68,69]. Furthermore, the PROTECT IV trial is currently enrolling HR-PCI patients with impaired left-sided heart function to assess whether pre-emptive Impella CP implantation, compared to current standard care, can lead to improvements in survival, MACCE, and quality of life [70,71]. Despite these ongoing efforts, there are still several uncertainties surrounding the optimal use of Impella devices. These include issues such as the patient selection criteria, the timing of pump placement, the role of Impella in promoting extensive coronary revascularization, the implementation of shock protocols, the management of DRC, and exploring new applications in conditions like transient LV dysfunction (e.g., takotsubo cardiomyopathy, peripartum cardiomyopathy, and spontaneous coronary dissection) or as support for off-pump coronary bypass grafts. While some of these questions have been addressed in observational studies, large-scale randomized evidence is needed to provide definitive answers [72,73].

## 7. Conclusions

As the Impella device continues to undergo widespread adoption in the management of acute MI complicated by CS and non-emergent CHIP, it is essential for clinicians to recognize the current state of the art and ongoing areas of investigation. While there is a strong physiological rationale for Impella use and some supporting data, the impact on long-term clinical and safety outcomes remains uncertain and conflicting. As such, further investigation is warranted to clarify its role in optimizing patient outcomes. In the absence of definitive evidence-based knowledge, several key factors become paramount for clinicians. These include multidisciplinary team agreement, meticulous patient selection, optimal PCI technique, operator experience, and standardized algorithms for pre- and post-procedural management. By prioritizing these elements, clinicians can navigate the complexities surrounding Impella use and contribute to advancing the field through careful and informed decision-making.

## Abbreviations and Acronyms (Alphabetical Order)

AKI	acute kidney injury
BCIS-JS	British Cardiovascular Intervention Society Myocardial Jeopardy Score
CAD	coronary artery disease
CHIP	complex high-risk indicated procedure
CS	cardiogenic shock
ECMO	extracorporeal veno-arterial membrane oxygenation
EF	ejection fraction
HR-PCI	high-risk PCI
IABP	intra-aortic balloon pump
LV	left ventricle
LVEDP	left ventricle end-diastolic pressure
MAE	major adverse event
MACE	major cardiovascular event
MACCEs	major adverse cardiac and cerebrovascular events
MCS	mechanical circulatory support
PCI	percutaneous coronary intervention
PCWP	pulmonary capillary wedge pressure
pLVAD	percutaneous left ventricular assist device
RCT	randomized controlled trial
SYNTAX	Synergy Between Percutaneous Coronary Intervention with Taxus and Cardiac Surgery

## References

[1] Thiele H., Zeymer U., Neumann F.-J., Ferenc M., Olbrich H.-G., Hausleiter J., Richardt G., Hennersdorf M., Empen K., Fuernau G. (2012). Intraaortic Balloon Support for Myocardial Infarction with Cardiogenic Shock. N. Engl. J. Med..

[2] Lüsebrink E., Kellnar A., Krieg K., Binzenhöfer L., Scherer C., Zimmer S., Schrage B., Fichtner S., Petzold T., Braun D. (2022). Percutaneous Transvalvular Microaxial Flow Pump Support in Cardiology. Circulation.

[3] Pahuja M., Johnson A., Kabir R., Bhogal S., Wermers J.P., Bernardo N.L., Ben-Dor I., Hashim H., Satler L.F., Sheikh F.H. (2022). Randomized Trials of Percutaneous Microaxial Flow Pump Devices. J. Am. Coll. Cardiol..

[4] Burkhoff D., Sayer G., Doshi D., Uriel N. (2015). Hemodynamics of Mechanical Circulatory Support. J. Am. Coll. Cardiol..

[5] Chieffo A., Dudek D., Hassager C., Combes A., Gramegna M., Halvorsen S., Huber K., Kunadian V., Maly J., Møller J.E. (2021). Joint EAPCI/ACVC expert consensus document on percutaneous ventricular assist devices. EuroIntervention.

[6] Chieffo A., Burzotta F., Pappalardo F., Briguori C., Garbo R., Masiero G., Nicolini E., Ribichini F., Trani C., Álvarez B.C. (2019). Clinical expert consensus document on the use of percutaneous left ventricular assist support devices during complex high-risk indicated PCI. Int. J. Cardiol..

[7] Vetrovec G.W., Anderson M., Schreiber T., Popma J., Lombardi W., Maini B., Moller J.E., Schäfer A., Dixon S.R., Hall S. (2018). The cVAD registry for percutaneous temporary hemodynamic support: A prospective registry of Impella mechanical circulatory support use in high-risk PCI, cardiogenic shock, and decompensated heart failure. Am. Heart J..

[8] O’Neill W.W., Kleiman N.S., Moses J., Henriques J.P., Dixon S., Massaro J., Palacios I., Maini B., Mulukutla S., Džavík V. (2012). A Prospective, Randomized Clinical Trial of Hemodynamic Support With Impella 2.5 Versus Intra-Aortic Balloon Pump in Patients Undergoing High-Risk Percutaneous Coronary Intervention. Circulation.

[9] Kovacic J.C., Kini A., Banerjee S., Dangas G., Massaro J., Mehran R., Popma J., O’Neill W.W., Sharma S.K. (2015). Patients with 3-Vessel Coronary Artery Disease and Impaired Ventricular Function Undergoing PCI with Impella 2.5 Hemodynamic Support Have Improved 90-Day Outcomes Compared to Intra-Aortic Balloon Pump: A Sub-Study of The PROTECT II Trial. J. Interv. Cardiol..

[10] Dangas G.D., Kini A.S., Sharma S.K., Henriques J.P., Claessen B.E., Dixon S.R., Massaro J.M., Palacios I., Popma J.J., Ohman E.M. (2013). Impact of Hemodynamic Support With Impella 2.5 Versus Intra-Aortic Balloon Pump on Prognostically Important Clinical Outcomes in Patients Undergoing High-Risk Percutaneous Coronary Intervention (from the PROTECT II Randomized Trial). Am. J. Cardiol..

[11] Cohen M.G., Matthews R., Maini B., Dixon S., Vetrovec G., Wohns D., Palacios I., Popma J., Ohman E.M., Schreiber T. (2015). Percutaneous left ventricular assist device for high-risk percutaneous coronary interventions: Real-world versus clinical trial experience. Am. Heart J..

[12] Burzotta F., Russo G., Ribichini F., Piccoli A., D’amario D., Paraggio L., Previ L., Pesarini G., Porto I., Leone A.M. (2019). Long-Term Outcomes of Extent of Revascularization in Complex High Risk and Indicated Patients Undergoing Impella-Protected Percutaneous Coronary Intervention: Report from the Roma-Verona Registry. J. Interv. Cardiol..

[13] Wollmuth J., Patel M.P., Dahle T., Bharadwaj A., Waggoner T.E., Chambers J.W., Ruiz-Rodriguez E., Mahmud E., Thompson C., Morris D.L. (2022). Ejection Fraction Improvement Following Contemporary High-Risk Percutaneous Coronary Intervention: RESTORE EF Study Results. J. Soc. Cardiovasc. Angiogr. Interv..

[14] Panoulas V.F., Escaned J., Hill J.M., Barker E., Butler K., Almedhychy A., Tsintzos S.I., O’neill W.W. (2024). Predictors of left ventricular ejection fraction in high-risk percutaneous coronary interventions. Front. Cardiovasc. Med..

[15] Chieffo A., Ancona M.B., Burzotta F., Pazzanese V., Briguori C., Trani C., Piva T., De Marco F., Di Biasi M., Pagnotta P. (2020). Observational multicentre registry of patients treated with IMPella mechanical circulatory support device in ITaly: The IMP-IT registry. EuroIntervention.

[16] Tarantini G., Masiero G., Burzotta F., Pazzanese V., Briguori C., Trani C., Piva T., De Marco F., Di Biasi M., Pagnotta P. (2021). Timing of Impella implantation and outcomes in cardiogenic shock or high-risk percutaneous coronary revascularization. Catheter. Cardiovasc. Interv..

[17] Flaherty M.P., Moses J.W., Westenfeld R., Palacios I., O’Neill W.W., Schreiber T.L., Lim M.J., Kaki A., Ghiu I., Mehran R. (2019). Impella support and acute kidney injury during high-risk percutaneous coronary intervention: The Global cVAD Renal Protection Study. Catheter. Cardiovasc. Interv..

[18] Azzalini L., Johal G.S., Baber U., Bander J., Moreno P.R., Bazi L., Kapur V., Barman N., Kini A.S., Sharma S.K. (2021). Outcomes of Impella-supported high-risk nonemergent percutaneous coronary intervention in a large single-center registry. Catheter. Cardiovasc. Interv..

[19] Lansky A.J., Tirziu D., Moses J.W., Pietras C., Ohman E.M., O’Neill W.W., Ekono M.M., Grines C.L., Parise H. (2022). Impella Versus Intra-Aortic Balloon Pump for High-Risk PCI: A Propensity-Adjusted Large-Scale Claims Dataset Analysis. Am. J. Cardiol..

[20] Buijs D.M.v.D., Wilgenhof A., Knaapen P., Zivelonghi C., Meijers T., Vermeersch P., Arslan F., Verouden N., Nap A., Sjauw K. (2022). Prophylactic Impella CP versus VA-ECMO in Patients Undergoing Complex High-Risk Indicated PCI. J. Interv. Cardiol..

[21] Schweitzer J., Horn P., Voss F., Kivel M., Wolff G., Jung C., Zeus T., Kelm M., Westenfeld R. (2021). Incidence of Acute Kidney Injury Is Lower in High-Risk Patients Undergoing Percutaneous Coronary Intervention Supported with Impella Compared to ECMO. J. Cardiovasc. Transl. Res..

[22] Baumann S., Werner N., Al-Rashid F., Schäfer A., Bauer T., Sotoudeh R., Bojara W., Shamekhi J., Sinning J.-M., Becher T. (2020). Six months follow-up of protected high-risk percutaneous coronary intervention with the microaxial Impella pump: Results from the German Impella registry. Coron. Artery Dis..

[23] McDonagh T.A., Metra M., Adamo M., Gardner R.S., Baumbach A., Böhm M., Burri H., Butler J., Čelutkienė J., Chioncel O. (2021). 2021 ESC Guidelines for the diagnosis and treatment of acute and chronic heart failure. Eur. Heart J..

[24] Wayangankar S.A., Bangalore S., McCoy L.A., Jneid H., Latif F., Karrowni W., Charitakis K., Feldman D.N., Dakik H.A., Mauri L. (2016). Temporal Trends and Outcomes of Patients Undergoing Percutaneous Coronary Interventions for Cardiogenic Shock in the Setting of Acute Myocardial Infarction. JACC Cardiovasc. Interv..

[25] Seyfarth M., Sibbing D., Bauer I., Fröhlich G., Bott-Flügel L., Byrne R., Dirschinger J., Kastrati A., Schömig A. (2008). A Randomized Clinical Trial to Evaluate the Safety and Efficacy of a Percutaneous Left Ventricular Assist Device Versus Intra-Aortic Balloon Pumping for Treatment of Cardiogenic Shock Caused by Myocardial Infarction. J. Am. Coll. Cardiol..

[26] Karami M., Eriksen E., Ouweneel D.M., E Claessen B., Vis M.M., Baan J., Beijk M., Packer E.J.S., Sjauw K.D., Engstrom A. (2021). Long-term 5-year outcome of the randomized IMPRESS in severe shock trial: Percutaneous mechanical circulatory support vs. intra-aortic balloon pump in cardiogenic shock after acute myocardial infarction. Eur. Heart J. Acute Cardiovasc. Care.

[27] Bochaton T., Huot L., Elbaz M., Delmas C., Aissaoui N., Farhat F., Mewton N., Bonnefoy E. (2019). Mechanical circulatory support with the Impella® LP5.0 pump and an intra-aortic balloon pump for cardiogenic shock in acute myocardial infarction: The IMPELLA-STIC randomized study. Arch. Cardiovasc. Dis..

[28] Møller J.E., Engstrøm T., Jensen L.O., Eiskjær H., Mangner N., Polzin A., Schulze P.C., Skurk C., Nordbeck P., Clemmensen P. (2024). Microaxial Flow Pump or Standard Care in Infarct-Related Cardiogenic Shock. N. Engl. J. Med..

[29] Hochman J.S., Sleeper L.A., Webb J.G., Sanborn T.A., White H.D., Talley J.D., Christopher E.B., Jacobs A.K., Slater J.N., Col J. (1999). Early Revascularization in Acute Myocardial Infarction Complicated by Cardiogenic Shock. N. Engl. J. Med..

[30] Thiele H., Zeymer U., Akin I., Behnes M., Rassaf T., Mahabadi A.A., Lehmann R., Eitel I., Graf T., Seidler T. (2023). Extracorporeal Life Support in Infarct-Related Cardiogenic Shock. N. Engl. J. Med..

[31] Masiero G., Cardaioli F., Tarantini G. (2022). Mechanical circulatory support in cardiogenic shock: A critical appraisal. Expert Rev. Cardiovasc. Ther..

[32] Schäfer A., Westenfeld R., Sieweke J.-T., Zietzer A., Wiora J., Masiero G., Martinez C.S., Tarantini G., Werner N. (2021). Complete Revascularisation in Impella-Supported Infarct-Related Cardiogenic Shock Patients Is Associated With Improved Mortality. Front. Cardiovasc. Med..

[33] Schrage B., Becher P.M., Bernhardt A., Bezerra H., Blankenberg S., Brunner S., Colson P., Deseda G.C., Dabboura S., Eckner D. (2020). Left Ventricular Unloading Is Associated With Lower Mortality in Patients With Cardiogenic Shock Treated With Venoarterial Extracorporeal Membrane Oxygenation. Circulation.

[34] Lemor A., Dehkordi S.H.H., Basir M.B., Villablanca P.A., Jain T., Koenig G.C., Alaswad K., Moses J.W., Kapur N.K., O’Neill W. (2020). Impella Versus Extracorporeal Membrane Oxygenation for Acute Myocardial Infarction Cardiogenic Shock. Cardiovasc. Revascularization Med..

[35] Schrage B., Ibrahim K., Loehn T., Werner N., Sinning J.-M., Pappalardo F., Pieri M., Skurk C., Lauten A., Landmesser U. (2019). Impella Support for Acute Myocardial Infarction Complicated by Cardiogenic Shock. Circulation.

[36] Dhruva S.S., Ross J.S., Mortazavi B.J., Hurley N.C., Krumholz H.M., Curtis J.P., Berkowitz A., Masoudi F.A., Messenger J.C., Parzynski C.S. (2020). Association of Use of an Intravascular Microaxial Left Ventricular Assist Device vs Intra-aortic Balloon Pump With In-Hospital Mortality and Major Bleeding Among Patients With Acute Myocardial Infarction Complicated by Cardiogenic Shock. JAMA.

[37] Wernly B., Karami M., Engström A.E., Windecker S., Hunziker L., Lüscher T.F., Henriques J.P., Ferrari M.W., Binnebößel S., Masyuk M. (2021). Impella versus extracorporal life support in cardiogenic shock: A propensity score adjusted analysis. ESC Heart Fail..

[38] Sieweke J.-T., Akin M., Beheshty J.-A., Flierl U., Bauersachs J., Schäfer A. (2021). Unloading in Refractory Cardiogenic Shock After Out-Of-Hospital Cardiac Arrest Due to Acute Myocardial Infarction—A Propensity Score-Matched Analysis. Front. Cardiovasc. Med..

[39] Scherer C., Lüsebrink E., Kupka D., Stocker T.J., Stark K., Stremmel C., Orban M., Petzold T., Germayer A., Mauthe K. (2020). Long-term clinical outcome of cardiogenic shock patients undergoing impella CP treatment vs. standard of care. J. Clin. Med..

[40] Karami M., den Uil C.A., Ouweneel D.M., Scholte N.T., Engström A.E., Akin S., Lagrand W.K., Vlaar A.P., Jewbali L.S., Henriques J.P. (2020). Mechanical circulatory support in cardiogenic shock from acute myocardial infarction: Impella CP/5.0 versus ECMO. Eur. Heart J. Acute Cardiovasc. Care.

[41] Redfors B., Watson B.M., McAndrew T., Palisaitis E., Francese D.P., Razavi M., Safirstein J., Mehran R., Kirtane A.J., Généreux P. (2017). Mortality, Length of Stay, and Cost Implications of Procedural Bleeding After Percutaneous Interventions Using Large-Bore Catheters. JAMA Cardiol..

[42] Ancona M.B., Montorfano M., Masiero G., Burzotta F., Briguori C., Pagnesi M., Pazzanese V., Trani C., Piva T., De Marco F. (2021). Device-related complications after Impella mechanical circulatory support implantation: An IMP-IT observational multicentre registry substudy. Eur. Heart J. Acute Cardiovasc. Care.

[43] O’Neill W.W., Anderson M., Burkhoff D., Grines C.L., Kapur N.K., Lansky A.J., Mannino S., McCabe J.M., Alaswad K., Daggubati R. (2022). Improved outcomes in patients with severely depressed LVEF undergoing percutaneous coronary intervention with contemporary practices. Am. Heart J..

[44] Leick J., Werner N., Mangner N., Panoulas V., Aurigemma C. (2022). Optimized patient selection in high-risk protected percutaneous coronary intervention. Eur. Heart J. Suppl..

[45] Pietrasik A., Gąsecka A., Jasińska-Gniadzik K., Szwed P., Grygier M., Pawłowski T., Sacha J., Kochman J. (2023). Roadmap towards an institutional Impella programme for high-risk coronary interventions. ESC Heart Fail..

[46] Tehrani B.N., Truesdell A.G., Sherwood M., Desai S., Tran H.A., Epps K.C., Singh R., Psotka M., Shah P., Cooper L.B. (2019). Standardized Team-Based Care for Cardiogenic Shock. J. Am. Coll. Cardiol..

[47] Basir M.B., Schreiber T.L., Grines C.L., Dixon S.R., Moses J.W., Maini B.S., Khandelwal A.K., Ohman E.M., O’Neill W.W. (2017). Effect of Early Initiation of Mechanical Circulatory Support on Survival in Cardiogenic Shock. Am. J. Cardiol..

[48] Leon S.A., Rosen J.L., Ahmad D., Austin M.A., Vishnevsky A., Rajapreyar I.N., Ruggiero N.J., Rame J.E., Entwistle J.W., Massey H.T. (2023). Microaxial circulatory support for percutaneous coronary intervention: A systematic review and meta-analysis. Artif. Organs.

[49] Sardone A., Franchin L., Moniaci D., Colangelo S., Colombo F., Boccuzzi G., Iannaccone M. (2023). Management of Vascular Access in the Setting of Percutaneous Mechanical Circulatory Support (pMCS): Sheaths, Vascular Access and Closure Systems. J. Pers. Med..

[50] Karatolios K., Hunziker P., Schibilsky D. (2021). Managing vascular access and closure for percutaneous mechanical circulatory support. Eur. Heart J. Suppl..

[51] Hayashida K., Lefèvre T., Chevalier B., Hovasse T., Romano M., Garot P., Mylotte D., Uribe J., Farge A., Donzeau-Gouge P. (2011). Transfemoral Aortic Valve Implantation. JACC Cardiovasc. Interv..

[52] Sandoval Y., Burke M.N., Lobo A.S., Lips D.L., Seto A.H., Chavez I., Sorajja P., Abu-Fadel M.S., Wang Y., Poulouse A. (2017). Contemporary Arterial Access in the Cardiac Catheterization Laboratory. JACC Cardiovasc. Interv..

[53] Ando T., Nakamaru R., Kohsaka S., Fukutomi M., Onishi T., Tobaru T. (2023). Access Site–Stratified Analysis of the Incidence, Predictors, and Outcomes of Impella-Supported Patients With Cardiogenic Shock. Am. J. Cardiol..

[54] Abdel-Wahab M., Hartung P., Dumpies O., Obradovic D., Wilde J., Majunke N., Boekstegers P., Müller R., Seyfarth M., Vorpahl M. (2022). Comparison of a Pure Plug-Based Versus a Primary Suture-Based Vascular Closure Device Strategy for Transfemoral Transcatheter Aortic Valve Replacement: The CHOICE-CLOSURE Randomized Clinical Trial. Circulation.

[55] van Wiechen M.P., Tchétché D., Ooms J.F., Hokken T.W., Kroon H., Ziviello F., Ghattas A., Siddiqui S., Laperche C., Spitzer E. (2020). Suture- or Plug-Based Large-Bore Arteriotomy Closure. JACC Cardiovasc. Interv..

[56] Verreault-Julien L., Shekiladze N., Wollmuth J., Rinfret S. (2022). Single-access for Impella-supported percutaneous coronary intervention using a sheathless technique with an 8 Fr guide. Catheter. Cardiovasc. Interv..

[57] Roberts N., Chandrasekaran U., Das S., Qi Z., Corbett S. (2020). Hemolysis associated with Impella heart pump positioning: In vitro hemolysis testing and computational fluid dynamics modeling. Int. J. Artif. Organs.

[58] Baldetti L., Beneduce A., Romagnolo D., Frias A., Gramegna M., Sacchi S., Calvo F., Pazzanese V., Cappelletti A.M., Ajello S. (2023). Impella Malrotation Within the Left Ventricle Is Associated With Adverse In-Hospital Outcomes in Cardiogenic Shock. JACC Cardiovasc. Interv..

[59] Balthazar T., Vandenbriele C., Verbrugge F.H., Uil C.D., Engström A., Janssens S., Rex S., Meyns B., Van Mieghem N., Price S. (2021). Managing Patients With Short-Term Mechanical Circulatory Support. J. Am. Coll. Cardiol..

[60] Van Edom C.J., Gramegna M., Baldetti L., Beneduce A., Castelein T., Dauwe D., Frederiks P., Giustino G., Jacquemin M., Janssens S.P. (2023). Management of Bleeding and Hemolysis During Percutaneous Microaxial Flow Pump Support. JACC Cardiovasc. Interv..

[61] Esposito M.L., Morine K.J., Annamalai S.K., O’kelly R., Aghili N., Pedicini R., Breton C., Mullin A., Hamadeh A., Kiernan M.S. (2018). Increased Plasma-Free Hemoglobin Levels Identify Hemolysis in Patients With Cardiogenic Shock and a Trans valvular Micro-Axial Flow Pump. Artif. Organs.

[62] Tarantini G., Mojoli M., Barioli A., Battistel M., Généreux P. (2016). Blood oozing: A cause of life-threatening bleeding without overt source after transcatheter aortic valve replacement. Int. J. Cardiol..

[63] Kormos R.L., Antonides C.F., Goldstein D.J., Cowger J.A., Starling R.C., Kirklin J.K., Rame J.E., Rosenthal D., Mooney M.L., Caliskan K. (2020). Updated definitions of adverse events for trials and registries of mechanical circulatory support: A consensus statement of the mechanical circulatory support academic research consortium. J. Heart Lung Transplant..

[64] Bertaina M., Galluzzo A., Rossello X., Sbarra P., Petitti E., Prever S.B., Boccuzzi G., D’Ascenzo F., Frea S., Pidello S. (2022). Prognostic implications of pulmonary artery catheter monitoring in patients with cardiogenic shock: A systematic review and meta-analysis of observational studies. J. Crit. Care.

[65] Atkinson T.M., Ohman E.M., O’neill W.W., Rab T., Cigarroa J.E. (2016). A Practical Approach to Mechanical Circulatory Support in Patients Undergoing Percutaneous Coronary Intervention. JACC Cardiovasc. Interv..

[66] Matassini M.V., Marini M., Angelozzi A., Angelini L., Shkoza M., Compagnucci P., Falanga U., Battistoni I., Pongetti G., Francioni M. (2023). Clinical outcomes and predictors of success with Impella weaning in cardiogenic shock: A single-center experience. Front. Cardiovasc. Med..

[67] Kapur N.K., Kim R.J., Moses J.W., Stone G.W., Udelson J.E., Ben-Yehuda O., Redfors B., Issever M.O., Josephy N., Polak S.J. (2022). Primary left ventricular unloading with delayed reperfusion in patients with anterior ST-elevation myocardial infarction: Rationale and design of the STEMI-DTU randomized pivotal trial. Am. Heart J..

[68] Delmas C., Laine M., Schurtz G., Roubille F., Coste P., Leurent G., Hraiech S., Pankert M., Gonzalo Q., Dabry T. (2023). Rationale and design of the ULYSS trial: A randomized multicenter evaluation of the efficacy of early Impella CP implantation in acute coronary syndrome complicated by cardiogenic shock. Am. Heart J..

[69] The RECOVER IV Trial-Full Text View-ClinicalTrials.gov. https://classic.clinicaltrials.gov/ct2/show/NCT05506449.

[70] The PROTECT-EU Study-Full Text View-ClinicalTrials.gov. https://classic.clinicaltrials.gov/ct2/show/NCT05466552.

[71] Study Details|Impella®-Supported PCI in High-Risk Patients with Complex Coronary Artery Disease and Reduced Left Ventricular Function|ClinicalTrials.gov. https://clinicaltrials.gov/study/NCT04763200.

[72] Tarantini G., Masiero G., Thiele H., Iannaccone M., Schrage B., Hassager C., Woitek F., Chieffo A., Møller J.E. (2023). Timing and treatment strategies according to SCAI classification in cardiogenic shock. Eur. Heart J. Suppl..

[73] Study Details|Protect Kidney Trial|ClinicalTrials.gov. https://clinicaltrials.gov/study/NCT04321148.

